# Tim-3^+^ decidual Mφs induced Th2 and Treg bias in decidual CD4^+^T cells and promoted pregnancy maintenance via CD132

**DOI:** 10.1038/s41419-022-04899-2

**Published:** 2022-05-12

**Authors:** Mengdie Li, Fengrun Sun, Yuanyuan Xu, Lanting Chen, Chunqin Chen, Liyuan Cui, Jinfeng Qian, Dajin Li, Songcun Wang, Meirong Du

**Affiliations:** Laboratory for Reproductive Immunology, Key Laboratory of Reproduction Regulation of NPFPC, SIPPR, IRD, Shanghai Key Laboratory of Female Reproductive Endocrine Related Diseases, Hospital of Obstetrics and Gynecology, Fudan University Shanghai Medical College, Shanghai, PR China

**Keywords:** Infertility, Translational immunology, Cell death and immune response

## Abstract

T-cell immunoglobulin mucin-3 (Tim-3) plays roles in the functional regulation of both adaptive and innate immune cells and is greatly involved in many diseases. However, the precise roles of Tim-3 on macrophages (Mφs) in pregnancy remain unstated. In the current study, we found the higher frequency of Tim-3^+^ decidual Mφs (dMφs) in response to trophoblasts. The reduced abundance of Tim-3 on Mφs was accompanied by disordered anti- and pro-inflammatory cytokine profiles in miscarriage. Adoptive transfer of Tim-3^+^Mφs, but not Tim-3^−^Mφs, relieved murine embryo absorption induced by Mφ depletion. Our flow cytometry results and the extensive microarray analysis confirmed that Tim-3^+^ and Tim-3^−^dMφs were neither precisely pro-inflammatory (M1) nor anti-inflammatory (M2) Mφs. However, with higher CD132 expression, Tim-3^+^dMφs subset induced Th2 and Treg bias in decidual CD4^+^T cells and promoted pregnancy maintenance. Blockade of Tim-3 or CD132 pathways leaded to the dysfunction of maternal-fetal tolerance and increased fetal loss. These findings underscored the important roles of Tim-3 in regulating dMφ function and maintaining normal pregnancy, and suggested that Tim-3 on Mφs is a potential biomarker for diagnosis of miscarriage. Our study also emphasized the importance of careful consideration of reproductive safety when choosing immune checkpoint blockade therapies in real world clinical care. Though IL-4 treated Tim-3^−^Mφs could rescue the fetal resorption induced by Mφ depletion, whether IL-4 represent novel therapeutic strategy to prevent pregnancy loss induced by checkpoint inhibition still needs further research.

## Introduction

Either impaired tolerance induction or excessive inflammation is believed to be associated with a lot of pregnancy-related complications, such as recurrent spontaneous abortion (RSA) [1]. Substantial research in reproductive and transplant immunology has been made to address the mechanism that the avoidance of immune attack by the semi-allogeneic fetus that has fetal histocompatibility antigens inherited from the father, while at the same time protecting the mother and the fetus from pathogens during pregnancy. For many years, Treg expansion and a polarization toward Th2 bias in the maternal immune response have long been considered the main mechanisms of inducing tolerance toward the fetus [2]. Now we believed that various kinds of cells, especially those residing at the maternal-fetal interface, play critical roles in this ‘immunological paradox’ of pregnancy [3].

The maternal-fetal interface is a highly dynamic tissue where the extravillous trophoblast cells (Tros) invade the decidua and come into direct contact with the decidual cells, and the appropriate crosstalk between each other is crucial for successful pregnancy. In early pregnancy, approximately 30–40% of total decidual cells are immune cells, of which macrophages (Mφs) are the most important specialized antigen presenting cells and the second most abundant leucocytes (10–20% of the decidual immune cells) at the maternal-fetal interface. The depletion of Mφs after conception has been reported to cause fetal resorption and embryo implantation arrest [4, 5], highlighting the importance of Mφs during pregnancy. Decidual Mφs (dMφs) are associated with the success of pregnancy by regulating immune responses and promoting decidual vascular remodeling [6]. Environmental cues and molecular mediators polarize Mφs as type 1 Mφs (M1) and type 2 Mφs (M2). M1 are more effective at antigen clearance and switching T cell responses toward Th1 immune response with the high expression of CD80, CD86, IL-12, and TNF-α. While M2 have more immunosuppressive capacities, contributing to tissue remodeling, and promoting Th2 immune responses with the high expression of CD163, CD206, CD209, and IL-10 [7]. Imbalance of M1/M2 leads to a pathological pregnancy, such as RSA [8].

T-cell immunoglobulin mucin-3 (Tim-3) is first identified as a specific cell surface marker of Th1 cells, and it mediates T cell exhaustion and apoptotic cell phagocytosis in chronic virus infection, organ transplantation and tumorigenesis. Later, Tim-3 was also found to be expressed on innate immune cells, such as Mφs [9]. However, the role of Tim-3 on Mφs is complex and controversial depending on distinct microenvironments in contrast to its well-recognized effects on T cell immunity. For example, it was reported that Tim-3 reduced the release of pro-inflammatory cytokines, such as IFN-γ and IL-12 by Mφs in inflammatory bowel disease and ameliorated the colitis [10]. While Tim-3 was also found to aggravated podocyte injury by promoting Mφs activation in diabetic nephropathy [11]. In pregnancy, Tim-3 is strikingly upregulated on monocytes in the peripheral blood compared to nonpregnant women [12]. In addition, Tim-3 blockade resulted in a decrease in phagocytic properties of dMφs leading to a failure to clear dead and apoptotic cells from the uterus [13].

We previously reported that downregulation or blockade of Tim-3 is associated with miscarriage by regulating T cell and NK cell functions [14, 15]. However, there are no studies demonstrating in full how Mφ Tim-3 works in the maternal-fetal immunity during early pregnancy. With all this information, we chose to determine the expression of Tim-3 on dMφs and to investigate its contribution to the maternal-fetal tolerance. In the present study, we found that the expression of Tim-3 was higher on dMφs in normal pregnancies than that in miscarriages. We also used normal pregnancy mouse model, abortion-prone mouse model, Mφ depletion and adoptive transfer experiments to determine the involvement of Mφ Tim-3 in pregnancy maintenance. Our findings showed evidence supporting the critical role of Tim-3 in inducing maternal-fetal tolerance by regulating dMφ functions and by regulating the crosstalk between dMφs and decidual CD4^+^T (dCD4^+^T) cells.

## Results

### Tros contributed to the higher proportion of Tim-3^+^CD14^+^ cells at the maternal-fetal interface

**Fig. 1:**
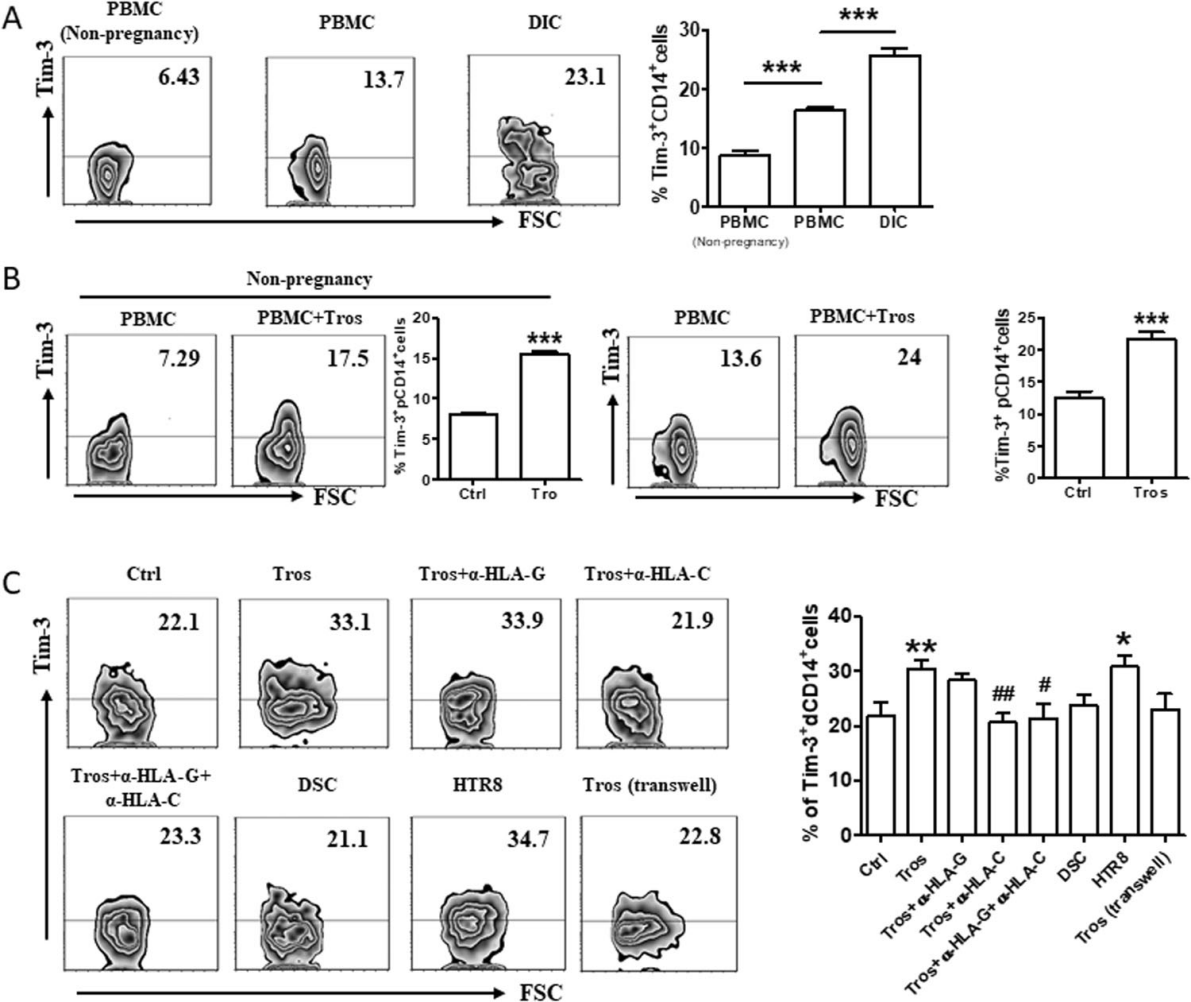
Expression of Tim-3 on CD14^+^ cells during human early pregnancy. **A** Flow cytometric analysis and quantification of frequency of Tim-3 expression on gated CD14^+^ cells from peripheral blood mononuclear cells (PBMCs) isolated from non-pregnant women of childbearing age (*n* = 9), and from paired PBMCs and decidual immune cells (DICs) during human first trimester pregnancy (*n* = 48). ***p < 0.001. **B** Quantification of flow cytometric analysis of Tim-3 expression on peripheral CD14^+^ (pCD14^+^) cells isolated from non-pregnant women or pregnant women, with or without co-culture with trophoblasts (Tros) for 48 h. *n* = 20. ***p < 0.001. **C** Quantification of flow cytometric analysis of Tim-3 expression on dCD14^+^ cells cultured alone or co-cultured with equal numbers of Tros (directly or indirectly), or decidual stromal cells (DSCs), or human HTR8/SVneo cells. The α-HLA-G/HLA-C antibodies were used in some wells. *p < 0.05, **p < 0.01, compared with the control. ^#^*p* < 0.05, ^##^*p* < 0.01, compared with the group co-cultured with Tros. Data represent the mean ± standard error of the mean (SEM). The flow cytometry plots are representative of three independent experiments.

We first compared the expression levels of Tim-3 on CD14^+^ cells from peripheral blood freshly isolated from non-pregnant women of childbearing age, and from paired decidua and peripheral blood from clinically normal first trimester pregnancies. Tim-3 was expressed on significantly higher proportions of peripheral monocytes (pMos) from pregnant women compared to that from nonpregnant women. While Tim-3 expression on dMφs was much higher than that on pMos during pregnancy (Fig. 1A).

Then we wanted to know why Tim-3 was highly expressed during pregnancy? The placenta has been regarded as a pseudo-malignant type of tissue due to the invasive property, high proliferation and immune escape capacity. As tumor micro-environment is responsible to the re-education of Mφs into tumor-associated Mφs [16], we established a co-culture system of Tros and pMos from non-pregnant or pregnant women. As shown in Fig. 1B, after 48 h, the frequency of Tim-3^+^ pMos increased, both in non-pregnant and pregnant women.

At the maternal-fetal interface, invading Tros, maternal-derived decidual stromal cells (DSCs), and decidual immune cells (DICs), come into direct contact with each other. We found that primary Tros and HTR8/Svneo cell line (an immortalized human extravillious trophoblast cell line), but not DSCs, could raise the expression of Tim-3 on dMφs. Separation of Tros and dMφs with a transwell insert eliminated the promotion of Tim-3 expression by Tros. The semi-allogeneic Tros have a unique human leukocyte antigen that expressing class I HLA-C and nonclassical HLA-G antigens [17]. Interestingly, administration of anti-HLA-C antibody, but not anti-HLA-G antibody, significantly inhibited Tros-induced up-regulation of Tim-3 expression on dMφs (Fig. 1C).

### Disorder of the number and function of Tim-3^+^dMφs in miscarriage

**Fig. 2:**
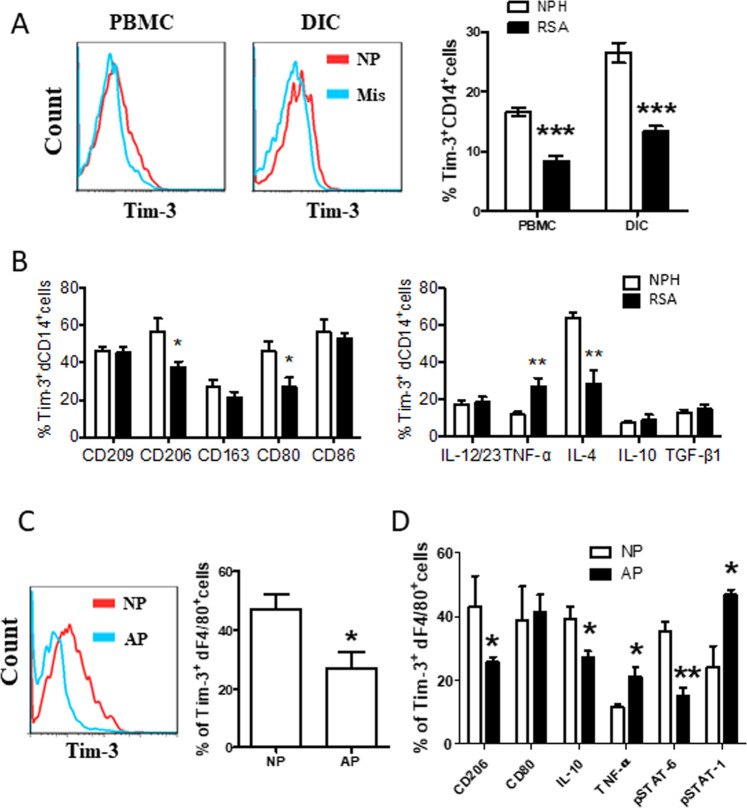
Decreased cell number of Tim-3^+^CD14^+^ cells with deficient function in miscarriage. **A** Frequency of Tim-3 expressing cells in gated CD14^+^ cells of paired PBMCs and DICs from normal pregnant subjects (NPH, normal pregnancy of human, *n* = 40) and patients who diagnosed as recurrent spontaneous abortion (RSA, *n* = 40) as determined by flow cytometric analysis. A representative dot plot is also shown. **B** Surface molecule expression and cytokine production by Tim-3^+^ decidual CD14^+^ (Tim-3^+^dCD14^+^) cells from normal pregnancy and RSA was assessed by flow cytometric analysis. Data represent the mean ± SEM. *P < 0.05, **P < 0.01. NPH: normal pregnancy of human, RSA: recurrent spontaneous abortion. **C** Frequency of Tim-3 expressing cells in gated F4/80^+^ cells from DICs of normal pregnant (NP, *n* = 17) and abortion-prone (AP, *n* = 18) mice. A representative dot plot is also shown. **D** Quantification of flow cytometric analysis of surface molecule expression and cytokine production by Tim-3^+^dF4/80^+^ cells from NP (*n* = 5) and AP (*n* = 4) mice. Data represent the mean ± SEM. *P < 0.05, **P < 0.01, ***P < 0.001. NP: normal pregnancy, AP: abortion prone. Data are representative of 4-5 independent analyses. *P < 0.05, **P < 0.01.

Next, we compared the frequency of Tim-3^+^CD14^+^ cells from normal pregnancy and RSA patients. We found that the number of Tim-3^+^CD14^+^ cells was lower in RSA patients than that form normal pregnancy, both in peripheral blood mononuclear cells (PBMCs) and DICs (Fig. 2A). Apart from the decreased frequency, Tim-3^+^dMφs in RSA patients expressed more TNF-α, but less CD206, CD80, IL-4 (Fig. 2B). We also established an abortion-prone model using female CBA/J x male DBA/2 mice, and observed similar phenomenon that decreased number and disordered function of Tim-3^+^dMφs in miscarriage (Fig. 2C).

### Blockade of Tim-3 led to dMφs dysfunction and murine fetal loss

**Fig. 3:**
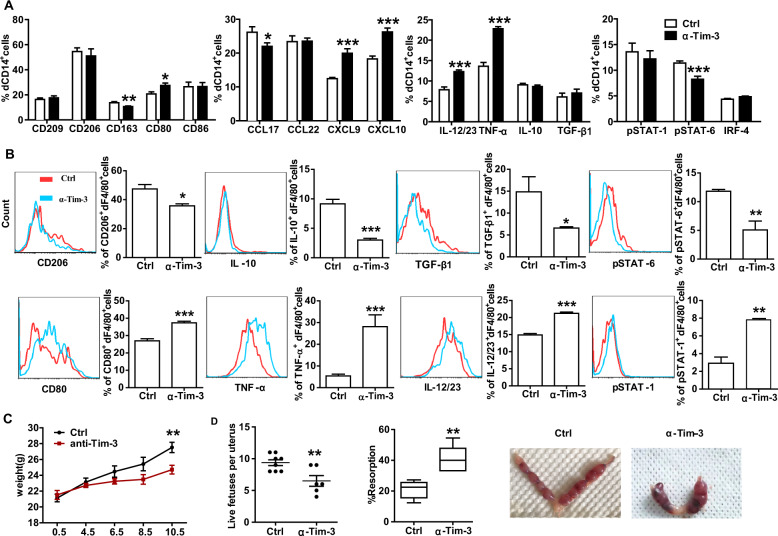
Blockade of Tim-3 led to dMφs dysfunction and murine fetal loss. **A** Quantification of flow cytometric analysis of surface molecule, cytokine and transcription factor expression by human dCD14^+^ cells cultured for 48 h in the presence or absence of anti-Tim-3 antibody (10 μg/ml). Data represent the mean ± SEM. *n* = 9. *P < 0.05, **P < 0.01, ***P < 0.001, compared with the control group. **B** Flow cytometric analysis and quantification of frequency of surface molecule, cytokine and transcription factor expression by dF4/80^+^ cells from pregnant CBA/J females following treatment with isotype IgG or anti-Tim-3 antibody i.p. at doses of 500, 250, and 250 mg at days 4.5, 6.5, and 8.5, respectively. **C** The weight of pregnant CBA/J females treated with isotype IgG or anti-Tim-3 antibody. **D** The number of live fetuses per uterus, the percent of fetal resorption and the representative images of uterus from pregnant CBA/J females treated with isotype IgG or anti-Tim-3 antibody. Data represent the mean ± SEM of *n* = 5–7 mice per group and are representative of three independent analyses. **p < 0.01, compared with the control group.

As the frequency and function of Tim-3^+^dMφs were both altered in miscarriage, we further tested whether blocking the Tim-3 pathway could change the functionalities of dMφs. In the first assay, expression levels of intracellular cytokines and transcription factors in human dMφs were analyzed. Compared with the control group, blockade of Tim-3 increased M1-associated CD80, CXCL9, CXCL10, IL-12/23 and TNF-α expression, but decreased M2-associated CD163 and CCL17 expression, and STAT-6 phosphorylation level in human dMφs (Fig. 3A).

In the second assay, we examined pregnant CBA/J females challenged with Tim-3 blocking antibody. Analysis of the dMφs from the treated mice revealed that CD206, IL-10, TGF-β1 expression and STAT6 phosphorylation level were decreased. While CD80, TNF-α, IL-12/23 expression and STAT-1 phosphorylation level were increased (Fig. 3B). Furthermore, treatment with Tim-3 blocking antibody caused decreased growth in body weight (Fig. 3C), reduction in the number of live fetuses per uterus, and a higher rate of embryo resorption (Fig. 3D). In addition, blockade of Tim-3 also increased the Th1-associated TNF-α, IFN-γ and T-bet, but decreased Th2-associated IL-4 and GATA-3 and Treg associated TGF-β1, IL-10 and Foxp3 expression by dCD4^+^T cells (Supplementary Fig. 1). Taken together with our in vitro data, Tim-3 pathway regulated immune responses in dMφs playing important role in the maintenance of maternal-fetal tolerance so to promote the establishment of normal pregnancy.

### Adoptive transfer of Tim-3^+^Mφs relieved murine embryo absorption induced by Mφ depletion

**Fig. 4:**
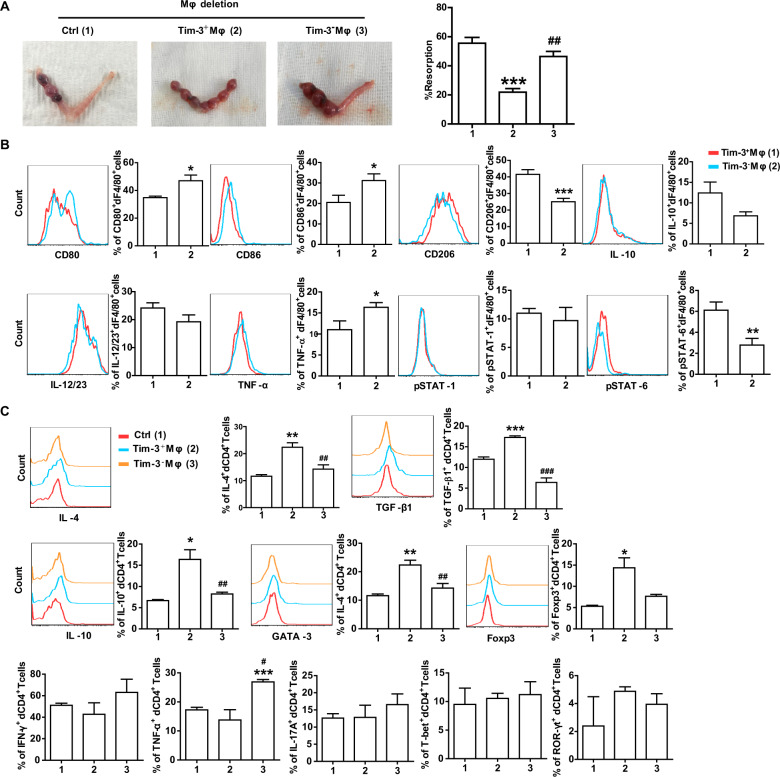
Adoptive transfer of Tim-3^+^Mφs relieved murine embryo absorption induced by Mφ depletion. **A** Representative images of uterus and the percent of fetal resorption in pregnant CBA/J mice with Mφ depletion or those that received adoptive transfer of Tim-3^+^Mφs or Tim-3^−^Mφs and at GD 4.5. Data represent the mean ± SEM of *n* = 4 mice per group and are representative of three independent analyses. ***p < 0.001, compared with the control group. ^##^*p* < 0.01, compared with the group of Tim-3^+^Mφs. **B** Flow cytometric analysis and quantification of frequency of surface molecule, cytokine and transcription factor expression by dF4/80^+^ cells from pregnant CBA/J females with Mφ depletion after adoptive transfer of Tim-3^+^Mφs or Tim-3^−^Mφs. Data represent the mean ± SEM of *n* = 6–9 mice per group. *p < 0.05, **p < 0.01, compared with the Tim-3^+^Mφs group. **C** Quantification of flow cytometric analysis of cytokine production and transcription factor expression by dCD4^+^ T cells from pregnant CBA/J females with Mφ depletion or those that received adoptive transfer of Tim-3^+^Mφs or Tim-3^−^Mφs. Data represent the mean ± SEM of *n* = 3–6 mice per group. *p < 0.05, **p < 0.01, ***p < 0.001, compared with the control group. ^#^*p* < 0.5, ^##^*p* < 0.01, ^###^*p* < 0.001, compared with the group of Tim-3^+^Mφs.

To provide direct insight into the role of Tim-3^+^Mφs in maternal-fetal immune regulation and pregnancy outcome in vivo, we evaluated the effect of Mφ depletion and adoptive transfer of Tim-3^+^Mφs on these processes. We isolated Tim-3^+^ and Tim-3^−^Mφs from pregnant mouse spleen and transferred them to Mφ-deleted pregnant mice. We found that adoptive transfer of Tim-3^+^Mφs, not Tim-3^−^Mφs, could significantly relieve the murine embryo absorption induced by Mφ depletion (Fig. 4A). To investigate the process of Tim-3^+^Mφs differentiation in the uterus in vivo, we labeled these Tim-3^+^ and Tim-3^−^Mφs with PKH-67 and transferred them to Mφ-deleted pregnant mice (Supplementary Fig. 2). In comparison with the PKH-67-Tim-3^−^Mφs transferred group, PKH-67-Tim-3^+^Mφs recruited to uterus presented lower levels of CD80, CD86 and TNF-α, higher levels of CD206, and an increase in STAT-6 activation (Fig. 4B).

In addition, the transfer of PKH-67-Tim-3^+^Mφs led to Th2 and Treg bias in dCD4^+^T cells in Mφ-deleted pregnant mice. While dCD4^+^T cells in the transfer of PKH-67-Tim-3^−^Mφs group expressed lower IL-4, TGF-β1, IL-10 and GATA-3, but higher TNF-α than that of PKH-67-Tim-3^+^Mφs transferred group (Fig. 4C). It seemed that Tim-3^+^Mφs and Tim-3^−^Mφs could differently regulate dCD4^+^T cell function during pregnancy. To confirm this conjecture, we further co-cultured human dCD4^+^T cells with Tim-3^+^dMφs or Tim-3^−^dMφs and analyzed the expression levels of intracellular cytokines and transcription factors in dCD4^+^T cells. As shown in Supplementary Fig. 3, Tim-3^+^dMφs increased Th2-associated IL-4, IL-13 and GATA-3, and Treg-associated IL-10, TGF-β1 and Foxp3 expression, but decreased Th1-associated IFN-γ expression in dCD4^+^T cells. While Tim-3^−^dMφs decreased IL-4, IL-13, GATA-3 and IL-10 expression in dCD4^+^T cells. Compared with Tim-3^−^dMφs, Tim-3^+^dMφs induced more Th2- and Treg-associated cytokines and transcription factors, but less IFN-γ expression in dCD4^+^T cells. Interestingly, neither Tim-3^+^dMφs nor Tim-3^−^dMφs had effect on TNF-α, IL-17A production and T-bet and ROR-γt expression by dCD4^+^T cells.

### Tim-3^+^dMφs and Tim-3^−^dMφs showed distinct CD132 expression indicative of different functional regulation on dCD4^+^T cells

**Fig. 5:**
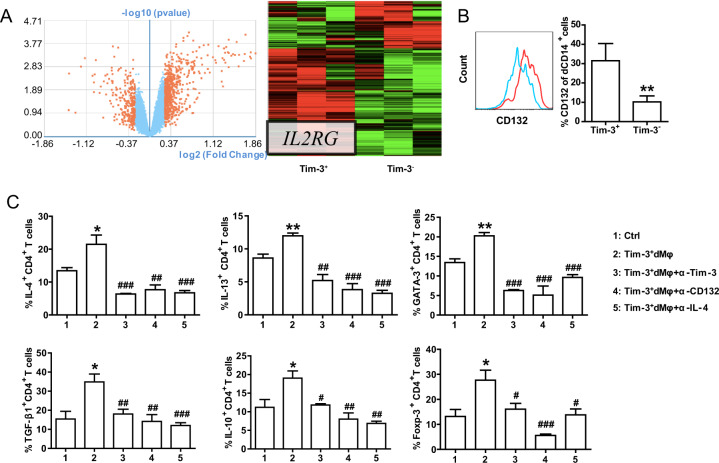
Tim-3^+^dMφs and Tim-3^−^dMφs showed distinct CD132 expression indicative of different functional regulation on dCD4^+^T cells. **A** The volcano plot and cluster analysis heat map of the differential mRNAs between Tim-3^+^dMφs and Tim-3^−^dMφs form human normal early pregnancy. Volcano plot based on fold change and *p* value of genes from the unique signatures of Tim-3^+^dMφs and Tim-3^−^dMφs in comparison with each other. Red dots mark the genes with significantly increased or decreased expression. Heatmap result of an unsupervised hierarchical clustering of genes that is significantly different in Tim-3^+^dMφs samples compared with Tim-3^−^dMφs samples. For example, gene encoding *IL2RG* was overexpressed in the Tim-3^+^dMφs population as compared with the Tim-3^−^dMφs. **B** Flow cytometric analysis and quantification of CD132 (the protein product of *IL2RG* gene) by human Tim-3^+^dMφs and Tim-3^−^dMφs (*n* = 9). **p < 0.01, compared with group of Tim-3^+^dMφs. The flow cytometry plots are representative of three independent experiments. **C** Flow cytometric analysis and quantification of Th2- and Treg-related cytokine expression by human dCD4^+^ T cells co-cultured with Tim-3^+^dMφs in the present or absence of anti-Tim-3 antibody, anti-CD132 antibody, or anti-IL-4 antibody. Data represent the mean ±SEM. *p < 0.05, **p < 0.01, compared with the control group. ^#^*p* < 0.5, ^##^*p* < 0.01, ^###^*p* < 0.001, compared with the group of Tim-3^+^dMφs.

We wanted to know why Tim-3^+^Mφs and Tim-3^−^Mφs played different roles in pregnant outcomes and dCD4^+^T cell function. We first compared the M1 and M2 markers of Tim-3^+^dMφs and Tim-3^−^dMφs by flow cytometry (FCM). Unexpectedly, Tim-3^+^dMφs expressed higher surface molecules, cytokines and transcription factors than Tim-3^−^dMφs, almost all the M1 and M2 markers (Supplementary Fig. 4A). Next, we conducted microarray profiling on highly purified populations isolated by FCM to understand what genomic differences distinguish these dMφs subsets. Unique gene signatures for Tim-3^+^Mφs and Tim-3^−^Mφs were generated based on 1.2-fold differential expression between the two different Mφ population. These commonly differentially expressed probes from Tim-3^+^dMφs and Tim-3^−^dMφs were highlighted on the combined data sets and presented as a volcano plot and a cluster analysis heat map (Fig. 5A). Are these Tim-3^+^dMφs and Tim-3^−^dMφs unique gene signatures common to other previously reported Mφs-derived gene expression profiles? Comparison with the published microarray analysis of M1 and M2 (Supplementary Fig. 4B) [18], neither Tim-3^+^ nor Tim-3^−^dMφs population can be categorized as strictly M1 or M2. And this was consistent with our FCM results (Supplementary Fig. 4A). While comparison with the published microarray analysis of dMφs and maternal peripheral monocytes (Supplementary Fig. 4C) [19] showed that the unique gene signature for Tim-3^+^dMφs correlated with the published dMφs, as there were 359 common mRNA differences, in which 302 were both upregulated in Tim-3^+^dMφ and dMφ (data not shown).

Gene signatures were composed of 669 probes upregulated specifically in the Tim-3^+^dMφs population and 294 probes downregulated in the Tim-3^−^dMφs. For example, genes encoding *IL2RG*, *EPAS1* and *M-CSF* were overexpressed in the Tim-3^+^dMφs population as compared with the Tim-3^−^dMφs. The protein product of *IL2RG* gene is a type I cytokine receptor known as the common gamma chain, which was also named CD132. CD132 is a cytokine receptor subunit that forms a complex with the ligand specific receptors for IL-2, IL-4, IL-7, IL-9, IL-15 and IL-21, to provide a common signaling chain for these receptors [20]. This aroused our interest as IL-4 was important for T cell tolerance while Tim-3^+^dMφs promoted Th2 and Treg bias in dCD4^+^T cells. Then we confirmed the differential expression of CD132 identified by this microarray analysis using FCM (Fig. 5B).

Was CD132 the reason that Tim-3^+^Mφs and Tim-3^−^Mφs differently regulate dCD4^+^T cell function during pregnancy? We co-cultured human dCD4^+^T cells with Tim-3^+^dMφs in the presence or absence of antibodies blocking Tim-3 or CD132 or IL-4. As shown in Fig. 5C, Tim-3^+^dMφs promoted Th2- and Treg-associated cytokines and transcription factors expression in dCD4^+^T cells. Pretreatment with anti-Tim-3 or anti-CD132 or anti-IL-4 antibody all could notably reverse the functional regulation of Tim-3^+^dMφs on dCD4^+^T cells. Thus Tim-3^+^dMφs and Tim-3^−^dMφs showed CD132 expressional differences indicative of unique function on regulating dCD4^+^T cell tolerance.

### Blockade of CD132 pathway counteracted protective effect of Tim-3^+^Mφs on murine pregnancy

**Fig. 6:**
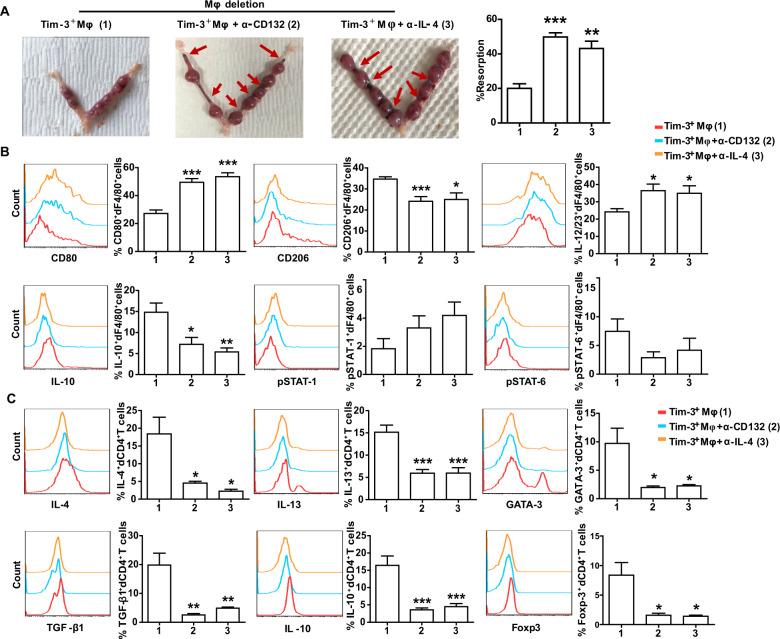
Blockade of CD132 pathway counteracted protective effect of Tim-3^+^Mφs on murine pregnancy. **A** Representative images of uterus and the percent of fetal resorption in pregnant CBA/J mice with Mφ depletion that received adoptive transfer of indicated Tim-3^+^Mφs. Fetal loss sites could be identified as hemorrhagic spots and necrosis (red arrows). **B** Flow cytometric analysis and quantification of frequency of surface molecule, cytokine and transcription factor expression by dF4/80^+^ cells from pregnant CBA/J females with Mφ depletion that received adoptive transfer of indicated Tim-3^+^Mφs. **C** Quantification of flow cytometric analysis of cytokine production and transcription factor expression by dCD4^+^ T cells from pregnant CBA/J females with Mφ depletion that received adoptive transfer of indicated Tim-3^+^Mφs. Data represent the mean ± SEM of *n* = 4–9 mice per group and are representative of three independent analyses. *p < 0.05, **p < 0.01, ***p < 0.001, compared with the Tim-3^+^Mφs group.

We further tested whether blocking the CD132 pathway could influence the murine pregnancy outcome of Tim-3^+^Mφs adoptive transfer. As shown in Fig. 6A, treatment with either anti-CD132 or anti-IL-4 antibody counteracted the protective effect of Tim-3^+^Mφs adoptive transfer on Mφ deleted murine pregnancy. Compared with Tim-3^+^Mφs transferred group, additional treatment with anti-CD132 or anti-IL-4 antibody caused greater susceptibility to fetal loss, leading to higher rate of embryo resorption. Does the more fetal loss of treated mice result from the dysfunction of dMφs under anti-CD132 or anti-IL-4 antibody treatment? Analysis of the dMφs from the treated mice revealed that CD80 and IL-23/23 expression of dMφs were increased, while CD206 and IL-10 expression were decreased (Fig. 6B). In addition, blockade of CD132 pathway also influenced dCD4^+^T cell tolerance as IL-4, IL-13, TGF-β1 and IL-10 production, and GATA-3 and Foxp-3 expression of dCD4^+^T cells were decreased. These data suggested that Tim-3^+^Mφs with the capacity to induce Th2 and Treg bias in dCD4^+^T cells via CD132 pathway contributed to maternal-fetal tolerance and pregnancy maintenance.

**Fig. 7:**
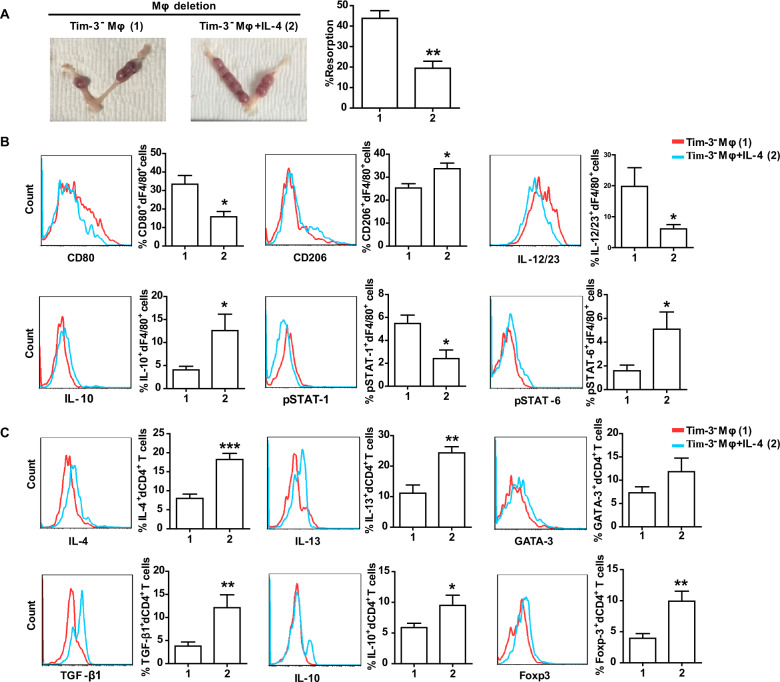
IL-4 treated Tim-3^−^Mφs also relieved murine embryo absorption induced by Mφ depletion. **A** Representative images of uterus and the percent of fetal resorption in pregnant CBA/J mice with Mφ depletion that received adoptive transfer of indicated Tim-3^−^Mφs. **B** Flow cytometric analysis and quantification of frequency of surface molecule, cytokine and transcription factor expression by dF4/80^+^ cells from pregnant CBA/J females with Mφ depletion that received adoptive transfer of indicated Tim-3^−^Mφs. **C** Quantification of flow cytometric analysis of cytokine production and transcription factor expression by dCD4^+^ T cells from pregnant CBA/J females with Mφ depletion that received adoptive transfer of indicated Tim-3^−^Mφs. Data represent the mean ± SEM of *n* = 6–9 mice per group. *p < 0.05, **p < 0.01, compared with the Tim-3^−^Mφs group.

While additional treatment with IL-4 in Tim-3^−^Mφs transferred group rescued the fetal resorption induced by Mφ depletion (Fig. 7A), which was concomitant with the increased M2-associated cytokines production by dMφs (Fig. 7B) and Th2- and Treg-associated cytokines production by dCD4^+^T cells (Fig. 7C). Thus IL-4 could also regulate Tim-3^−^Mφ function through other receptors at the same time.

## Discussion

RSA is a prevalent and distressing disorder that defined as two or more consecutive pregnancy losses prior to 20 weeks of gestation. The psychological and physical burden on the women is often unacceptable, and it can be compounded that with each loss they experience. However, progress in predicting and preventing RSA has been hampered by the uncertainties surrounding the pathogenesis. It is well recognized that either impaired tolerance induction or excessive inflammation is believed to be associated with RSA [1]. In humans, dMφs may be involved in Tros invasion, vascular remodeling, and the development of maternal-fetal tolerance [6]. In the present study, we supplied evidence that Tim-3 was a key regulator of dMφ function and therefore played important roles in the maintenance of normal pregnancy.

Increased evidence demonstrated the dynamic expression and diverse functions of Tim-3 in different disease models, especially in tumors, infections and autoimmune diseases [21–23]. Our present study demonstrated that Tim-3 expression in dMφs was significantly increased in normal pregnancy and acted as a modulator in the maintenance of normal pregnancy. This increased expression might due to local microenvironment at the maternal-fetal interface. On the basis of direct contact, embryonic Tros contributed to the increased Tim-3 expression on dMφs in an HLA-C-restricted manner. However, DSCs had no effect on the Tim-3 expression on dMφs, further confirming the importance of Tros in the establishment of maternal-fetal tolerance that maternal immune cells could be educated by embryonic Tros to develop a unique phenotype and tolerate the fetus [24, 25]. The number and function of Tim-3^+^dMφs in the decidua were significantly impaired in miscarriage, and adoptive transfer of Tim-3^+^Mφs, not Tim-3^−^Mφs relieved murine embryo absorption induced by Mφ depletion also suggesting that the expression of Tim-3 on dMφs during pregnancy might conduce to the maintenance of maternal-fetal immune tolerance and normal pregnancy.

Tim-3 not only works as a checkpoint of T cell activation but also plays important roles in Mφ functions by regulating cell activation, cytokine release, and phagocytic property, contributing to the progress of various diseases [13, 21]. Using Tim-3-blocking antibody, we clearly showed that Tim-3 suppressed anti-inflammatory cytokines of dMφs both in vivo and in vitro. We also found that pro-inflammatory cytokines of dCD4^+^T cells was elevated after the addition of anti-Tim-3 antibody. Furthermore, pregnant CBA/J females treated with Tim-3-blocking antibodie became more susceptible to fetal loss, although the further development of any surviving embryos needs additional study. Blockade of Tim-3 to improve immune cell responses is considered as novel strategies for the treatment of tumors and chronic infections [26, 27]. However, with the potential of birth defects and unknown risks of harm to mothers, and use of Tim-3 blockade agents in pregnancy would likely pose great risk of abortion, reproductive safety would ultimately be an individualized decision made with careful consideration of potential benefits and risks.

Mφs have remarkable plasticity allowing them to respond efficiently to varying environmental stimuli, and dMφs have always been identified as M2 in normal pregnancy that abnormally activated dMφs can disrupt the tolerance at the maternal-fetal interface [6]. However, mounting evidence indicated that initial classification scheme was an over-simplification of dMφs [28, 29]. Though Tim-3^+^Mφs led to Th2 and Treg bias in dCD4^+^T cells and Tim-3^−^Mφs decreased IL-4, IL-13, GATA-3 and IL-10 expression in dCD4^+^T cells, our FCM results and extensive microarray analysis further confirmed that Tim-3^+^ nor Tim-3^−^dMφs were neither precisely pro-inflammatory (M1) nor anti-inflammatory (M2) Mφs. In addition, FCM results showed that Tim-3^+^dMφs produced more inflammatory cytokines, as well as anti-inflammatory cytokines than Tim-3^+^dMφs. This finding seemed in contrast with the notion that the fetal-maternal interface is an anti-inflammatory environment, but fits more with the hypothesis that immune activation is required to facilitate Tros invasion and establishment of fetal-maternal tolerance. Interestingly, the cytokine expression in the whole dMφs treated with anti-Tim-3 antibody were not the same as that in Tim-3^−^dMφs. Blockade of Tim-3 even increased the expression of some M1-associated markers in the whole dMφs. Anti-Tim-3 antibody might be not sufficient enough to block all the Tim-3 signal in dMφs, so dMφs treated with anti-Tim-3 antibody were not the same as Tim-3^−^dMφs. Whether anti-Tim-3 antibody effects other pathways that regulating M1 and M2 differentiation still needs further study. However, these results further confirmed that Mφs have remarkable plasticity allowing them to respond efficiently to varying environmental stimuli.

Further analysis revealed that *IL2RG* gene were overexpressed in the Tim-3^+^dMφs population as compared with the Tim-3^−^dMφs. The protein product of *IL2RG* gene is CD132, also known as the common gamma chain. CD132-dependent cytokines, including IL-2, IL-4, IL-7, IL-9, IL-15, and IL-21, play crucial roles in the proliferation, survival, and differentiation of multiple cell lineages of both the innate and adaptive immune systems [20]. Tim-3^+^dMφs and Tim-3^−^dMφs showed CD132 expressional differences resulting in unique function on regulating dCD4^+^T cell tolerance. In addition, blockade of CD132 pathway counteracted the protective effect of Tim-3^+^Mφs on maternal-fetal tolerance and murine pregnancy.

IL-4 is a CD132-dependent cytokine known to activate the Jak-STAT pathway [30], which is important for functional regulation of CD4^+^T cells and Mφs. Level of IL-4 increases throughout normal pregnancy [31]. Administration of IL-4 could decrease the resorption rate in abortion prone mice [32]. Deficiency of IL-4 induced systemic and placental inflammation in mice and low IL-4 level was found in women with RSA [31]. Our present study demonstrated that additional treatment Tim-3^+^Mφs with anti-IL-4 antibody caused higher rate of embryo resorption than that in the adoptive transfer group alone. Furthermore, dysfunction of dMφs and dCD4^+^T cells by the production of disordered cytokines maybe associated with the fetal loss induced by additional anti-IL-4 antibody treatment. While treatment with IL-4 in Tim-3^−^Mφs transferred group rescued the fetal resorption induced by Mφ depletion. As CD132 expression was much lower in Tim-3^−^Mφs, IL-4 might also play regulatory role through other receptors that expressed on dMφs at the same time. In fact, previous report demonstrated that anti-inflammatory activities of IL-4 can be attributed to the direct action of this cytokine on myeloid effector cells, depending on their expression of the IL-4 receptor alpha chain CD124 [33].

**Fig. 8:**
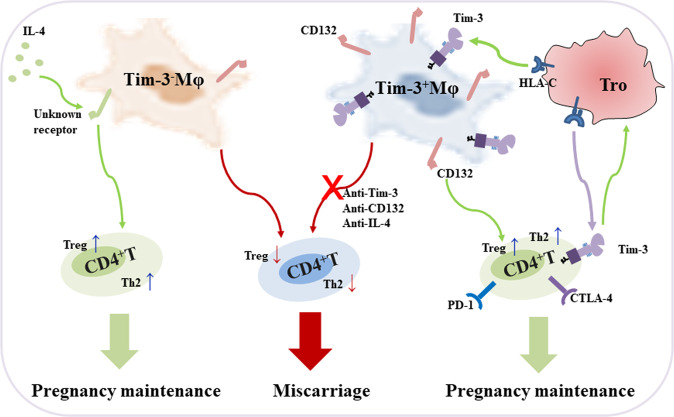
Schematic diagram of functional regulation of Tim-3^+^Mφs on maternal-fetal tolerance. Previously we reported that Tros directly contributed to the Tim-3, PD-1 and CTLA-4 expression on dCD4^+^T cells. These checkpoint pathways, in turn, may operate within the functional immune-modulatory network not only to promote maternal-fetal tolerance but also improved Tros function. The current study demonstrated that Tros also induced the higher Tim-3 expression on dMφs during normal pregnancy in HLA-C dependent manner. Neither Tim-3^+^ nor Tim-3^−^dMφs population can be categorized as strictly M1 or M2. With higher CD132 expression, Tim-3^+^dMφs induced Th2 and Treg bias in dCD4^+^T cells and promoted pregnancy maintenance. The reduced abundance of Tim-3 on Mφs was accompanied by disordered anti- and pro-inflammatory cytokine profiles in miscarriage. Blockade of Tim-3 or CD132 pathways leaded to the dysfunction of maternal-fetal tolerance and increased fetal loss. While IL-4 treated Tim-3^−^Mφs, via unknown receptor, could rescue the fetal resorption induced by Mφ depletion.

Collectively, our data describe the potential role of Tim-3 on Mφs as an inducer of maternal-fetal tolerance in pregnancy (Fig. 8). We identified a distinct subset of dMφs, Tim-3^+^dMφs, with immune-regulatory activity during normal pregnancy. With higher CD132 expression, Tim-3^+^dMφs induced Th2 and Treg bias in dCD4^+^T cells and promoted pregnancy maintenance. The reduced abundance of Tim-3 on Mφs was accompanied by disordered anti- and pro-inflammatory cytokine profiles in miscarriage. Blockade of Tim-3 or CD132 pathways leaded to the dysfunction of maternal-fetal tolerance and increased fetal loss. IL-4 treated Tim-3^−^Mφs could rescue the fetal resorption induced by Mφ depletion. These findings underscored the important roles of Tim-3 in regulating dMφ function and maintaining normal pregnancy, and suggested that Tim-3 on Mφs is a potential biomarker for diagnosis of miscarriage. Previously we also reported that Tros contributed to promote Tim-3、programmed cell death-1 (PD-1) and cytotoxic T-lymphocyte-associated protein 4 (CTLA-4) expression on dCD4^+^T cells. These checkpoint pathways, in turn, might operate within the functional immune-modulatory network not only to promote maternal-fetal tolerance but also to improve Tros function through DICs-Tros interaction dependent on IL-4 and IL-10 [14, 34, 35]. Whether IL-4 represent novel therapeutic strategy to prevent pregnancy loss induced by checkpoint inhibition still needs further research. However, combined with our present study, we believed that reproductive safety must be considered when checkpoint inhibitors were used during pregnancy, though it was reported that conception and viable twin pregnancy in a metastatic melanoma patient treated with CTLA-4 and PD-1 inhibitors [36].

## Materials and methods

### Ethical approval

This study was approved by the Research Ethics Committee of the Obstetrics and Gynecology Hospital, Fudan University (No. Kyy2017-50). Every participant signed a written informed consent form. All of animals were conducted in accordance with the National Guidelines for Animal Care and Use in Research (China). The experimental methods in particular were carried out in accordance with the approved guidelines.

### Human samples

Whole peripheral blood, villous and decidual tissues of human first-trimester pregnancies were obtained from clinically normal pregnancies (terminated for non-medical reasons, had at least one successful pregnancy and no history of spontaneous abortions, *N* = 86) and miscarriages (diagnosed as recurrent spontaneous abortion, RSA, and excluding those resulting from endocrine, anatomic, genetic abnormalities, infection, etc., *N* = 40). Whole peripheral blood samples were also obtained from normal non-pregnant women of childbearing age (*N* = 9).

PBMCs were isolated from peripheral blood samples using Ficoll density gradient centrifugation (Huajing, China). Tros were isolated by trypsin-DNase I (Applichem, Germany) digestion and discontinuous Percoll gradient centrifugation from the villus tissues as described previously [14]. DICs and DSCs were obtained from the decidual tissue digesting in RPMI 1640 (HyClone, U.S.A) supplemented with collagenase type IV (1.0 mg/ml, CLS-1, Worthington Biomedical, U.S.A) and DNase I (150 U/ml, Applichem, Germany) as described previously [14]. CD14^+^ cells were isolated by magnetic affinity cell sorting using CD14 microbeads (MiltenyiBiotec, Germany). Tim-3^+^Mφs and Tim-3^−^Mφs were isolated by BD FACSAria^TM^ III Cell Sorter using FITC-conjugated anti-human CD14 antibody, PE-conjugated anti-human Tim-3 antibody. CD4^+^ T cells were isolated by magnetic affinity cell sorting using CD4 microbeads (MiltenyiBiotec, Germany) or by BD FACSAria^TM^ III Cell Sorter using FITC-conjugated anti-human CD4 antibody.

### Cell Treatment

Freshly isolated Tros were seeded at a density of 2 × 10^5^ cells/ml per well in Matrigel (Coring, U.S.A)-coated 24-well plates overnight. The cells were then washed twice with phosphate-buffered saline (PBS, HyClone, U.S.A). Equal numbers of dCD14^+^ cells or pCD14^+^ cells were added to each well. In some wells, anti-HLA-C (10 μg/ml, clone W6/32; Biolegend, U.S.A), HLA-G (10 μg/ml, clone 87 G; Biolegend, U.S.A) were added. dCD14^+^ cells were also cultured with HTR8/Svneo cells (ATCC, www.atcc.org) or DSCs for 48 h. In some wells, dCD14^+^ cells (2 × 10^5^ cells) were plated in the upper chamber (0.4 mm pore size cell culture inserts, Millipore, Germany), while Tros were plated in the lower chamber to establish indirect cell contact.

Freshly isolated dCD4^+^ T cells co-cultured with Tim-3^+^dMφs or Tim-3^−^dMφs at a 1: 1 ratio. In some experiments, Tim-3^+^dMφs were pretreated with anti-Tim-3 (10 μg/ml, clone F38-2E2, BioLegend, U.S.A.), or anti-CD132 (10 μg/ml, clone TUGh4, BioLegend, U.S.A.), or anti-IL-4 (10 μg/ml, clone F38-2E2, BioLegend, U.S.A.). Phorbol 12-myrstate 13-acetate (PMA) (50 ng/ml, Biolegend, U.S.A.), ionomycin (1 μg/ml, Biolegend, U.S.A.) and brefeldin A (10 mg/ml, BioLegend, U.S.A.), were added 4 h before the end of the 48 h co-culture. The supernatants were then collected for intracellular cytokine analysis of T cells.

### RNA-Seq data analysis

The total RNA of sorted Tim-3^+^dMφs or Tim-3^−^dMφs was lysed and extracted according to the protocol of the RNeasy Mini Kit (Qiagen, Germany). Purity and integrity of the extracted RNA was tested by an Agilent Bioanalyzer 2100 (Agilent Technologies, U.S.A.). The prepared libraries were sequenced on an Illumina Hi-seq 2500 platform. Sequenced reads were aligned to the human reference genome using the STAR software package. Exons from all gene isoforms were merged to create one meta-gene. The number of reads falling in the exons of this meta-gene was counted using HTSeq-count, and differential expression analysis was conducted using DE-Seq. *P* < 0.05 were considered as the significance threshold [28, 37].

### Mice

CBA/J female, DBA/2 male, and BALB/c male mice were purchased from Slac laboratory animal Co. (Shanghai, China) and Huafukang bioscience Co. (Beijing, China) and maintained in an animal facility according to institutional and National Institutes of Health Guidelines. Eight-week-old CBA/J females were mated to BALB/c males to induce normal pregnancy (NP). Eight-week-old CBA/J females were mated to DBA/2 males to establish abortion-prone (AP) models. All the CBA/J females were inspected every morning for vaginal plugs. The day of visualization of a plug was designated as day 0.5 of pregnancy (GD 0.5). Some pregnant females of NP received injections of anti-Tim-3 antibody (clone RMT3-23, Biolegend, U.S.A.) or isotype IgG i.p. at doses of 500, 250, and 250 mg on days 4.5, 6.5, and 8.5, respectively based on our previous publication [14].

For Mφ depletion and Mφ adoptive transfer in pregnant CBA/J mice, Clodronate Liposomes were injected intraperitoneally at day 0.5 (200 μl) and day 3.5 (100 μl) of gestation. Tim-3^+^Mφs or Tim-3^−^Mφs were isolated from the spleen of C57BL/6 mice with an NP (GD 7.5) by BD FACSAria^TM^ III Cell Sorter and labeled with PKH-67 or not. The sorted cells were then resuspended in 200 ml of PBS and injected into the tail vein of Mφ-depleted pregnant mice at GD4.5. In some groups, Tim-3^+^Mφs were pre-stimulated with anti-CD132 (10 μg/ml, clone TUGm2, BioLegend, U.S.A.) or anti-IL-4 (10 μg/ml, clone 11B11, BioLegend, U.S.A.), Tim-3^−^Mφs were pre-stimulated with IL-4 (10 ng/ml, PeproTech, U.S.A) for 48 h. Pregnant mice were monitored at GD10.5. The percentage of fetal loss (the embryo absorption rate) was calculated as following: % of resorption = R/(R + V) × 100, where R represents the number of hemorrhagic implantation (sites of fetal loss) and V stands for the number of viable, surviving fetuses.

Uteri from pregnant mice were dissected free from the mesometrium and removed by cuts at the ovaries and cervix. The fetal and placental tissues were carefully removed and washed in PBS. Minced uteri were digested in RPMI 1640 supplemented with collagenase type IV and DNase I for 45–60 min at 37 °C with gentle agitation. Cells were cultured in RPMI 1640 supplemented with 10% FBS, 100 U/ml penicillin, 100 μg/ml streptomycin, and 1 μg/ml amphotericin B at 37 °C in 5% CO_2_ for 4 h to remove adherent stromal cells. The spleen was aseptically excised and stored in RPMI 1640. A single-cell suspension was made by using a 10-mL syringe plunger to pass spleen tissue into fresh wash medium through a 70-μm mesh strainer. PMA (50 ng/ml, Biolegend, U.S.A.), ionomycin (1 μg/ml, Biolegend, U.S.A.) and brefeldin A (10 mg/ml, BioLegend, U.S.A.), were added 4 h for intracellular cytokine analysis of T cells.

### Flow cytometry

Cell surface molecular expression and intracellular cytokine production were evaluated using flow cytometry. FITC-conjugated anti-human CD14, CD4, anti-mouse F4/80, CD4, TNF-α, IFN-γ, eFluor® 488-conjugated anti-human Foxp3, anti-mouse Foxp3, TNF-α, IFN-γ, STAT-1 Phospho, PE-conjugated anti-human Tim-3, CD132, IL-17A, IL-10, T-bet, GATA-3, anti-mouse CD80, Tim-3, TGF-β1, IL-10, IL-13, T-bet, PE/CY7-conjugated anti-human CD4, TNF-α, TGF-β1, anti-mouse IL-10, IL-12/23, TNF-α, T-bet, F4/80, PerCP/Cy5.5-conjugated anti-mouse T-bet, IL-17A, APC-conjugated anti-human Tim-3, IFN-γ, IL-13, ROR-γt, Foxp3, anti-mouse F4/80, TNF-α, Tim-3, IL-10, ROR-γt, GATA-3, STAT-6 Phospho, Pacific Blue-conjugated anti-mouse STAT-1 Phospho, Brilliant Violet 421-conjugated anti-human IL-4, anti-mouse CD206, TGF-β1, IL-4, TNF-α, STAT-1 Phospho, Brilliant Violet 510-conjugated anti-mouse CD4, CD86, TNF-α, IFN-γ, Brilliant Violet 605-conjugated anti-mouse CD4, IL-17A (Biolegend, U.S.A.), and PerCP-eFluor-710-conjugated anti-mouse GATA-3, (eBioscience, San Diego, CA, USA) antibodies were used. For intracellular staining, cells were fixed and permeabilized using the Fix/Perm kit (Biolegend, U.S.A.). Flow cytometry was performed on a Beckman-Coulter CyAn ADP cytometer (Beckman-Coulter, U.S.A.) and analyzed with FlowJo software (Tree Star, Ashland, U.S.A.).

### Statistical analysis

All variables were normally distributed in this study. Thus, variables were presented as means and standard deviation (SD). One-way analysis of variance (ANOVA) was used to evaluate differences. A *p* value of less than 0.05 was considered statistically significant. For variables with a *p* value of less than 0.05 in ANOVA, the post-hoc Dunnett *t*-test was performed to determine differences between each group. All analyses were carried out using the GraphPad Prism 8 software (GraphPad, San Diego, CA).

## Supplementary information

Supplementary files

## Data Availability

All data generated or analyzed during this study are included in the main text and the supplementary information files.
