# Supplementary material for: Tim-3+ decidual Mφs induced Th2 and Treg bias in decidual CD4+T cells and promoted pregnancy maintenance via CD132
Source: Cell Death Dis. 2022 May 12;13(5):454. doi: 10.1038/s41419-022-04899-2 (PMC9098864; doi:10.1038/s41419-022-04899-2)
Supplement: Supplementary file 1 — Supplementary files [file 41419_2022_4899_MOESM1_ESM.docx]

**
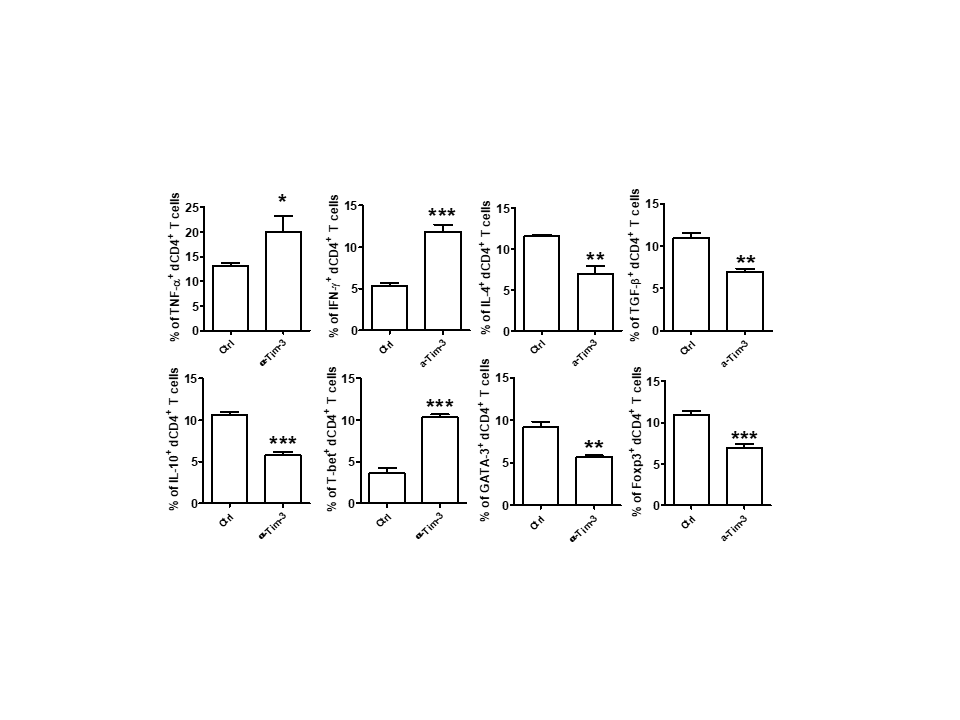
**

**Figure S1.** Quantification of flow cytometric analysis of cytokines production and transcription factors expression by dCD4^+^ T cells pregnant CBA/J females following treatment with isotype IgG or anti-Tim-3 antibody i.p. at doses of 500, 250, and 250 mg at days 4.5, 6.5, and 8.5 respectively. n=3-6. Data represent the mean ± SEM. *p<0.05, **p<0.01, ***p<0.001.


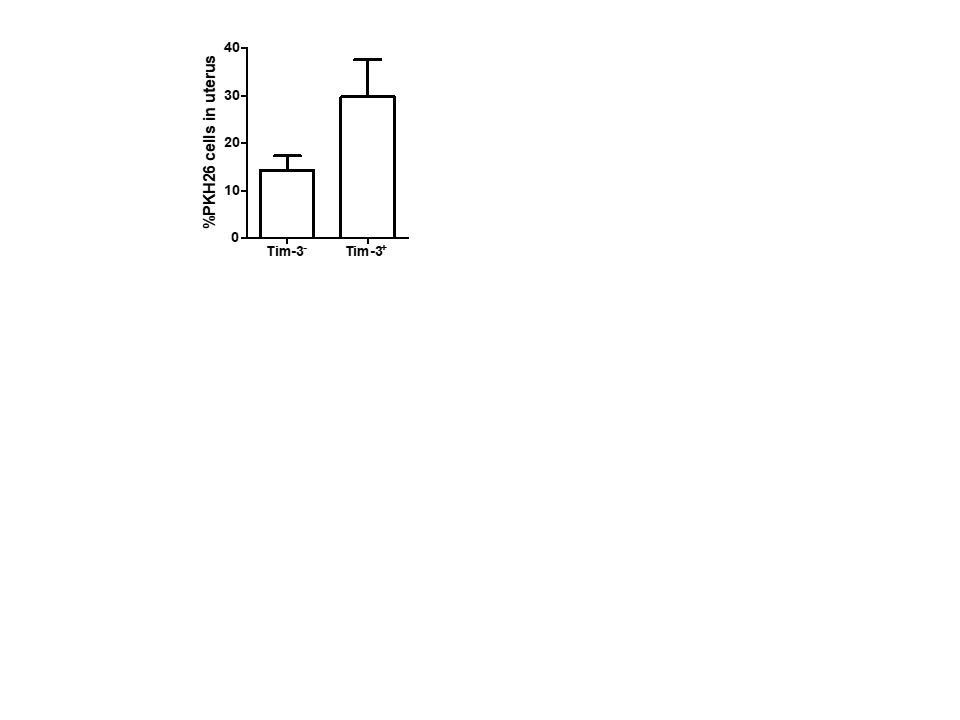


**Figure S2.** Flow cytometric identification of PKH-67-Tim-3^+^Mφ and PKH-67-Tim-3^-^Mφ transfer in pregnant mice uterus (n=3 mice/group) at GD10.5.

**
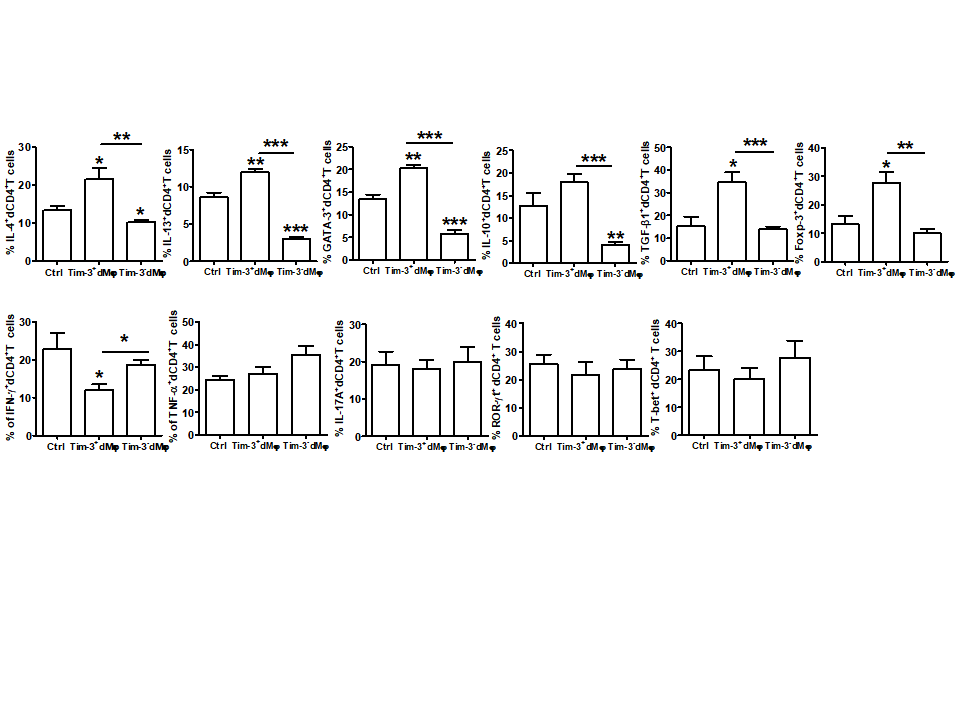
**

**Figure S3.** Quantification of flow cytometric analysis of cytokines production and transcription factors expression by human dCD4^+^ T cells co-cultured with Tim-3^+^dMφs or Tim-3^-^dMφs. Data represent the mean ± SEM. *p<0.05, **p<0.01, ***p<0.001.


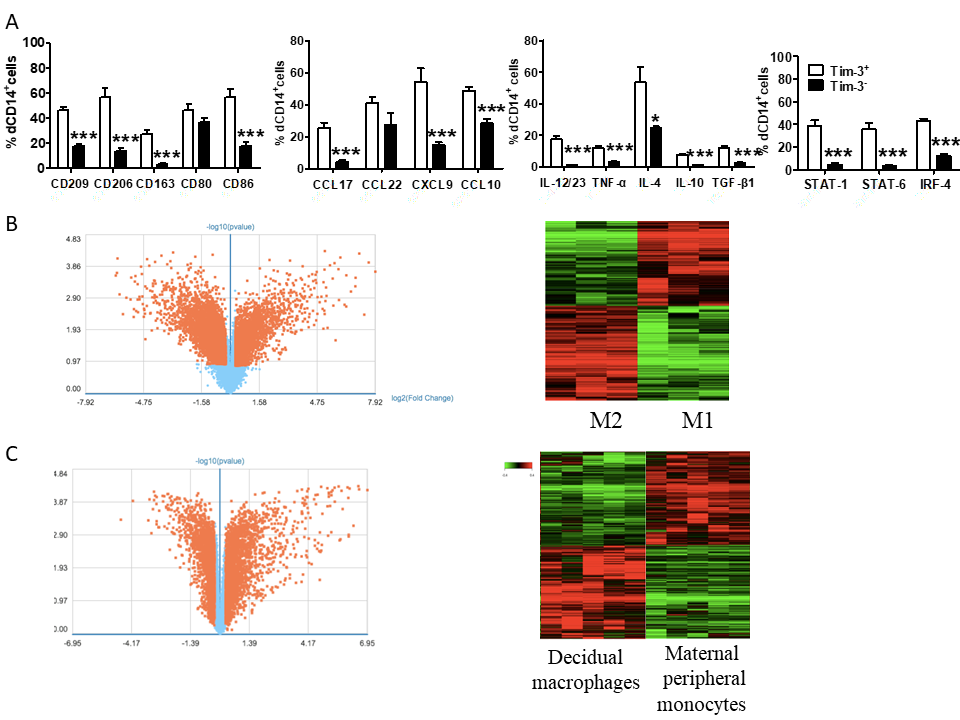


**Figure S4.** (A) Flow cytometric analysis and quantification of frequency of surface molecule, cytokine and transcription factor expression by Tim-3^+^dMφs or Tim-3^-^dMφs from the first trimester of human normal pregnancy. (B) The volcano plot and cluster analysis heat map of published data set that compared M1- and M2-derived Mφs (reference 18). Unique gene signatures from Tim-3^+^dMφs and Tim-3^-^dMφs were compared with data sets from M1- and M2-derived Mφs. There were 359 common mRNA differences, however, the trend of differential mRNA expression is not consistent on the whole. (C) The volcano plot and cluster analysis heat map of published data set that compared dMφ and maternal peripheral monocytes (reference 19). Comparison with the published microarray analysis of dMφs and maternal peripheral monocytes showed that the unique gene signature for Tim-3^+^dMφs correlated with the published dMφs, as there were 359 common mRNA differences, in which 302 were both upregulated in Tim-3^+^dMφ and dMφ.
